# Impact of the clinically oriented roles of a general practice receptionist: a systematic review with narrative synthesis

**DOI:** 10.3399/BJGP.2024.0228

**Published:** 2025-02-11

**Authors:** Keigo Ban, Sheila Greenfield, Michael Burrows, Nicola Gale, Ian Litchfield

**Affiliations:** Department of Applied Health Research, College of Medicine and Health, University of Birmingham, Birmingham.; Department of Applied Health Research, College of Medicine and Health, University of Birmingham, Birmingham.; Department of Forensic Psychology, School for Health and Life Sciences, Coventry University, Coventry.; Health Services Management Centre, School of Social Policy, University of Birmingham, Birmingham.; Department of Applied Health Research, College of Medicine and Health, University of Birmingham, Birmingham.

**Keywords:** access, general practice, medical receptionist, non-clinical roles, primary health care

## Abstract

**Background:**

Modern general practice is characterised by increased demand and growing multidisciplinarity, including ring-fenced funding for additional non-clinical roles. For practice receptionists, however, training has remained unchanged for decades despite primary care being under greater pressure than ever, with receptionists becoming a growing focal point for abuse and unprecedented numbers leaving the role.

**Aim:**

To present the evidence of the range of tasks that receptionists continue to perform, describing their impact on primary care delivery and how the role might be better supported.

**Design and setting:**

Systematic review of research conducted in the UK.

**Method:**

A systematic review of evidence contained in the major medical databases (MEDLINE/PubMed, CINAHL, ASSIA, Cochrane Library, and Embase) from January 2000 to March 2024 was conducted, including hand searches of the bibliographies of included studies.

**Results:**

In total, 29 studies were identified that grouped into three themes: service delivery, patient attitudes, and receptionist experience. The theme ‘service delivery’ confirms the continuing role of receptionists in providing administrative support alongside the clinical tasks of prioritising patients for consultations, facilitating repeat prescriptions, and communicating blood test results. The theme ‘patient attitudes’ describes how patients lacked trust in receptionists, who were viewed as unqualified and unnecessarily obstructive. Finally, in considering receptionist experience, the contrast between their confidence in performing administrative roles and the anxiety induced from the clinically related tasks was described, particularly the mounting pressure from patients to meet their preferences for clinician appointments.

**Conclusion:**

Although confident performing administrative tasks, receptionists described uncertainty and anxiety when providing clinically oriented support or managing patients when their requests for appointments could not be met. More appropriate training or professionalisation might improve staff retainment.

## Introduction

In 2024 the role of general practice receptionist in the UK is still being advertised as if they were working in a non-clinical office-based environment, that is, a job suitable for an individual with ‘people skills’ and ‘preferably some IT knowledge’.^1^ The historical description, expectation, and understanding of their role as administrators by patients, clinicians, and commissioners^2^^,^^3^ runs contrary to NHS England’s (NHSE) expectations of their place in supporting clinical care, involving tasks such as repeat prescribing, communicating test results, and prioritising patients’ clinical appointments.^4^^,^^5^ In UK primary care the unique physical location of the receptionist at the forefront of the practice premises and their contribution to multiple clinical and non-clinical processes means they have a key role to play in the equitable, integrated, and responsive NHS envisioned by policymakers.^4^^,^^6^

The post COVID-19 surge in patient demand in the UK^7^ highlighted the importance of receptionists in ensuring general practice operated safely and effectively.^8^ However, despite the prominence of their role, recent policy initiatives looking to improve access to primary care, including broadening the range of non-clinical roles, have failed to explicitly acknowledge the importance of receptionists.^9^^–^^11^ Its apparent underestimation by policymakers and senior decision-makers amid unprecedented demand has left them uniquely vulnerable to patient frustration.^2^^,^^12^^–^^16^ With their role in the provision of safe and equitable care seemingly unsupported, turnover of reception staff is higher than at any point since records began,^17^^,^^18^ at a time when NHSE are attempting to expand the roles of the non-clinical workforce.^19^

If primary care is to improve the retention of reception staff, then it is important to understand the scope of their role in modern primary care, including an exploration of their clinically relevant roles and the implications of such for patients, the service, and receptionists themselves that is currently lacking.^20^ In this way the negative influences on job satisfaction and self-efficacy can be mitigated, and appropriately targeted support and training provided.^20^ To achieve this the systematic review presented here is the first, to the authors’ knowledge, to collate research conducted in the UK exploring the work of GP receptionists.^20^ A narrative synthesis of the data is presented in this article drawing out key themes, and then practical and effective suggestions are presented to improve job satisfaction and autonomy in reception staff.

**Table:** How this fits in

There is growing attention on the impact of additional non-clinical members of the primary care team on safe and effective operation of general practice yet receptionists, the longest standing non-clinical role in general practice, continue to operate without the explicit recognition of receptionist roles. Despite the importance of the role of receptionists on the quality and safety of care, and patient experience, this systematic review is the first, to the authors’ knowledge, to explore their role in UK general practice. This review confirms the part receptionists play as key non-clinical members of the practice team who also perform a series of clinically related tasks; how patients can lack trust in their judgement and view them as unnecessarily obstructive; and that prioritising patients for access induces anxiety in receptionists.

## Method

### Study design

This review follows best practice as outlined by the *Cochrane Handbook for Systematic Reviews of Interventions*.^21^ The search history was reported following the PRISMA guideline,^22^ and the data described using a narrative synthesis. Further details are contained in the published protocol registered with Prospero (ID: CRD42016048957).^20^

### Eligibility criteria

The SPIDER tool (Sample, Phenomenon of Interest, Design, Evaluation, Research type) was used for developing eligibility criteria, as summarised in Box 1.^23^

**Box 1.** Representation of eligibility criteria using the SPIDER tool

**Components of SPIDER tool**	**Include**	**Exclude**
**S**ample	GP receptionists and other administrative jobs that regularly interact with patients	Employment without interaction with patients
**P**henomenon of **I**nterest	The general administrative and clinical roles of GP receptionists or relevant administrative jobs in general practices in the UK	Acute and emergency departments, specialist clinics and hospitals, or receptionists working in healthcare environments external to the UK
**D**esign	Any study design, that is, individual or focus group interviews, ethnography, controlled trials, or survey	None
**E**valuation	Studies that described GP receptionists’ roles without any outcomes or effects as well as those that described the consequences (for example, for the service or patient)	None
**R**esearch type	Primary and empirical studies including qualitative, quantitative, and mixed-method designs	Literature reviews and editorials without observation Studies not written in English Studies published before 2000

### Search strategy

The electronic databases MEDLINE/PubMed, CINAHL, ASSIA, Cochrane Library, and Embase were referred to. In addition, relevant individual journals (*British Journal of General Practice*, *British Medical Journal*, *Journal for Health Care Quality*, and *BMJ Quality and Safety*) were hand searched, and the bibliographies of the included studies were also screened. The search strategy was constructed in line with the SPIDER tool and performed from January 2000 to March 2024 (reflecting the modern era of general practice), summarised in Supplementary Table S1.

### Study selection

The first author performed title screening and studies with irrelevant titles were excluded; where there was uncertainty the first author consulted with the senior author. Studies with relevant titles proceeded to abstract screening, and those not meeting the criteria were excluded. The first and senior authors conducted a full-text review of the remaining studies and assessed the eligibility. Articles were included after agreement between the two was reached.

### Quality assessment and data extraction

Three different tools were used for the quality assessment process. The Critical Appraisal Skills Programme Qualitative Studies Checklist,^24^ the Quality Assessment Tool for Quantitative Studies,^25^ and the Mixed Methods Appraisal Tool^26^ were used for qualitative, quantitative, and mixed-method design, respectively. (The results of the quality assessment for each article are summarised in Supplementary Tables S2A, S2B, and S2C). The first and the senior author independently assessed the quality of each included study and discussions were held to address disagreements. Research measured as being of poor quality by the tools were excluded. The first and senior author extracted data including publication information, study characteristics, participant information, and outcomes. The references were managed using Endnote 20.

### Analysis

The narrative synthesis followed best practice, rigorously exploring relationships in the data within and between studies, and iteratively refining its interpretation to arrive at the three themes used to describe the evidence.^27^ This process was initially conducted independently by the first author and senior author beginning with the identification of more descriptive themes that summarised the data contained in each study, the authors then met to discuss these and worked together to refine these over time to produce the three consensually agreed themes within which the data are presented.

## Results

### Study selection

**Figure 1.**
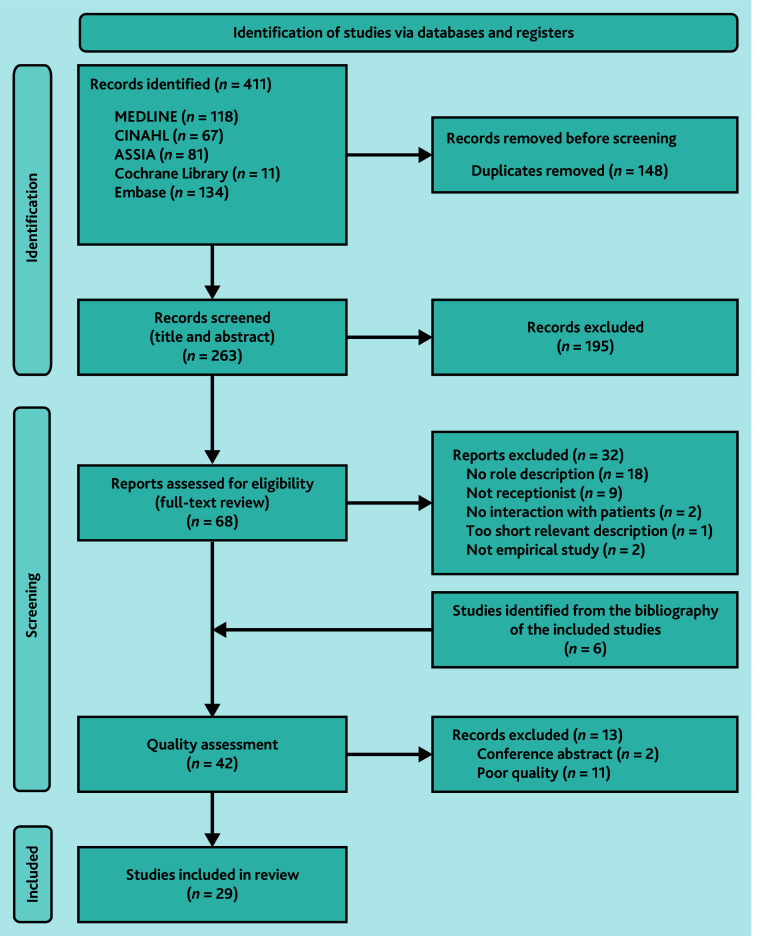
PRISMA flow diagram of the studies included in the review.

In total, 263 studies were identified after removing duplicates; of these, 195 studies were excluded following title and abstract screening. Two conference abstracts and 12 poor-quality studies were excluded at the quality assessment stage, with 29 studies included in the final review.^10^^,^^28^^–^^55^ The study selection process is shown in the PRISMA diagram (Figure 1).

### Characteristics of included articles

The characteristics of the included articles are summarised in Supplementary Table S3. The 29 studies identified were published between 2000 and 2023, six were quantitative,^28^^,^^29^^,^^32^^,^^34^^,^^39^^,^^43^ three were mixed methods,^30^^,^^33^^,^^53^ and the remaining 20 were qualitative.^10^^,^^31^^,^^35^^–^^38^^,^^40^^–^^42^^,^^44^^–^^52^^,^^54^^,^^55^ Among the six quantitative studies, five were cross-sectional surveys and one was a controlled trial.^39^ Of the 19 qualitative studies, six were ethnographies (including combinations with interviews),^38^^,^^44^^,^^48^^,^^50^^,^^54^^,^^55^ and 14 used individual or focus group interviews.^10^^,^^31^^,^^35^^–^^37^^,^^40^^–^^42^^,^^45^^–^^47^^,^^49^^,^^51^^,^^52^ In assessing the quality of the articles, 24 of 29 studies were assessed moderate and five were strong.^28^^,^^38^^,^^40^^,^^44^^,^^54^

Alongside receptionists, study participants included patients, GPs, and practice managers. The results are described within three themes that were iteratively determined by the focus dictated by the overall aim of each study. Specifically, these were:

- service delivery, describing the functions performed and the impact on patient care;
- patient attitudes, which presents their views on their interaction with receptionists and perceptions of their performance; and
- receptionist perspectives, which describes their overall understanding, and experience of the role.

#### Service delivery.

Studies confirmed receptionists continued to perform their recognised administrative tasks including compiling and filing medical letters, a range of office-related duties, and booking non-urgent appointments.^29^^,^^30^ This also included the associated task of directing patients to alternative sources of care and support.^31^^,^^32^ One study described how there was training for the signposting of a chronic condition, that is, skin complaints.^33^

However, there was evidence that routine appointment booking could assume the characteristics of triage when demand outstripped available appointments, often leaving receptionists responsible for prioritising patients based on judgements of clinical necessity.^10^^,^^34^^–^^38^^,^^40^ Three studies described how specific training was provided in this instance related to specific acute issues: domestic violence^41^^,^^42^ and stroke.^43^ Despite the apparent clinical implications of this aspect of their role, evidence from two articles described how GPs continued to consider these extensions of the appointment booking task as non-clinical.^31^^,^^32^

The role of receptionists in communicating blood test results was reported as a key element of timely diagnosis and/or the prognosis of patients.^28^^,^^29^^,^^44^^–^^47^ Reception staff were also responsible for facilitating repeat prescriptions with implications for the accuracy, timeliness, and safety of the process.^29^^,^^42^^,^^44^^,^^48^^–^^50^

#### Patient attitudes.

Receptionists studies described how patient attitudes were shaped by the burden involved in seeking an appointment, which typically required multiple attempts at contacting the practice via telephone before discovering the shortage of urgent appointments with the GPs.^51^^,^^52^ Once reaching practice staff, receptionists studies described how patients were reluctant to divulge clinical information^38^^,^^46^^,^^53^ and how they lacked trust in receptionist decision-making capabilities.^38^^,^^40^ The level of this distrust extended to patients believing receptionists hid available appointments to protect GPs,^10^^,^^37^ and that ‘care navigation’ was not about optimising access but solely about denying contact with GPs.^31^

Regarding the receptionists’ role in delivering test results, one study described how patients believed receptionists were capable of reporting test results,^45^ in two others the patients felt it inappropriate for receptionists to do so, describing their anxiety at being provided with no further information beyond that they needed to speak to a GP.^46^^,^^47^

#### Receptionist experience.

The majority of studies exploring the experiences of receptionists focused on their role in booking appointments where they described the stress and challenge when failing to meet patient expectations for urgency or seeing their preferred clinician.^28^^,^^29^^,^^31^^,^^38^^,^^40^^,^^54^ They also described the negotiations and conflict that could ensue when determining the nature and urgency of the patient request.^10^^,^^40^

However, one study did recognise the sensitivity of their role in dealing with concerned patients and reported the delivery of guidance to demonstrate empathy.^55^ Receptionists also described how their role in facilitating repeat prescribing was beyond their skill set and engendered anxiety and frustration,^42^^,^^49^^,^^50^ particularly when seeking GP sign off.^50^ Receptionists also described how communicating blood test results could cause anxiety and uncertainty, especially when left to deliver abnormal results.^45^^,^^46^ All of the above contrasted with their attitude to the non-clinical roles they performed where receptionists felt a high level of autonomy,^28^^,^^29^ although there were concerns over the threat of automation.^30^

## Discussion

### Summary

The role of receptionists is under increasing scrutiny as general practice organisations struggle to meet patient demand and increase the range of non-clinical roles. This review confirms the pivotal role receptionists play in delivering safe and effective clinical care often without recognition or relevant support.

Regarding their impact on service delivery, the continuing role of receptionists in performing a range of administrative tasks was confirmed, as were more explicit clinically related tasks, that is, prioritising patients for consultations, facilitating repeat prescriptions, and communicating blood test results. Studies exploring patient attitudes predominantly described the lack of trust in reception staff who were viewed as unnecessarily obstructive when attempting to gain access to GPs. Finally, in considering receptionist experience, receptionists described the contrast between their confidence in performing administrative roles and the anxiety induced from the clinically related tasks, particularly where judged by patients as the key decision-makers in allocating appointments.

### Strength and limitations

Notably this is the first systematic review, to the authors’ knowledge, exploring the roles and impact of GP receptionists. It provides a valuable summary of their value at a time when delivering primary care is complicated by a chronic lack of capacity, and non-clinical roles are being used to expand the primary care team.^18^ Best practice in review methodology was used, including data gathering, quality assessment, and analysis.^21^^,^^24^^–^^27^ Although a formal meta-analysis was not possible, the narrative synthesis enabled identification of three themes, which allowed a holistic interpretation of receptionist work. It also offered valuable insight into the toll taken by some of the blurred boundaries that persist in their role in the practice team.

The search was limited to beginning in January 2000 because of an awareness of the changes in size and scope of primary care in the past two decades that would have reduced the relevance of prior work conducted at a time when practices were much smaller and the primary care landscape less complicated. Although studies were limited to the UK, broader lessons can be drawn for medical office staff in other models of healthcare delivery where their interaction with clinical tasks is not explicitly defined and supported.^56^

### Comparison with existing literature

In terms of service delivery, the responsibility of receptionists for booking appointments has remained unchanged for decades yet is now performed in an environment of excessive demand and growing patient expectations.^57^^–^^59^ Although there have been changes in telecommunication technologies^60^ and referral pathways, the studies identified in the current study described how receptionists typically perform the role without structured support.^10^^,^^34^^–^^38^^,^^40^ It is notable that primary care receptionists perform similar clerical roles alongside tasks involving patient assessment in Australia,^61^^–^^64^ New Zealand,^65^ France,^66^ and the US.^67^

Any decision on the appropriate next step for patients made by reception staff is complicated by the increased options provided by the growing scale and scope of practice organisations^68^ and the sharing of resources across primary care networks^69^ alongside broader infrastructural shifts via the introduction of integrated care systems.^6^^,^^70^ This complexity also risks compromising receptionists’ contextual knowledge of individual patients gained from their continuity of contact.^71^ In the UK the receptionist workforce is predominantly White and female,^28^^,^^29^ which may be of note considering evidence that frontline public sector workers are more likely to demonstrate leniency towards requests of those with whom they share social demographic characteristics.^72^ However, this has yet to be explored in general practice receptionists.^73^

The pandemic motivated introduction of ‘total triage’ that required every patient seeking clinical contact to provide reasons and symptoms for their request and this was the first time the vital role of reception staff in triage was formally recognised, although it was mandated without additional training for any practice staff involved including receptionists.^60^^,^^74^^,^^75^ The introduction of online symptom checkers is expected to provide consistent algorithmic-based decisions on triaging patients and ease pressure on reception staff^76^ but with concerns that such automated systems will lose valuable contextual information.^77^^,^^78^

In terms of patient attitudes, the data described how adverse interactions with patients typically stemmed from their frustrations in obtaining a consultation.^31^^,^^51^^,^^52^ Issues in seeking appointments via telephone have existed for decades^59^^,^^79^ and NHSE have failed to substantively address the issue by staggering the release of appointments.^60^ Fundamentally the disconnect remains between a shrinking clinical workforce and an older, increasingly unhealthy population,^71^ meaning many patients with genuine needs either blame receptionists for a lack of access or fail to seek care.^17^ In other countries, such as New Zealand, receptionists can be viewed with similar suspicion, particularly among underserved populations,^80^ and in Australia receptionists experience similar abusive and intimidatory behaviours.^15^

Evidence from this review described the mistrust patients have in receptionists because of their lack of clinical training^45^^,^^46^ or the suspicion that they are dishonest brokers focused on protecting GPs.^38^^,^^53^ It also means that similar to other frontline care providers they can be exploited by patients gaming the system who view them as less consequential than their clinical colleagues.^81^^,^^82^

Patients’ hesitation in disclosing their symptoms to receptionists might be mitigated by the mooted professionalisation of GP receptionists.^38^^,^^83^ In the meantime, more must be done to educate patients around the concept that non-clinical intermediaries, such as receptionists or those employed through the Additional Roles Reimbursement Scheme (ARRS), are capable of supporting the most appropriate course of care and/or support.^84^

Finally, in terms of the receptionist experience, the studies identified described how their involvement in repeat prescribing and communicating test results led to uncertainty and anxiety among receptionists.^29^^,^^42^^,^^45^^–^^47^^,^^49^^,^^50^ However, it was their role in providing access that appeared particularly problematic,^28^^,^^29^^,^^31^^,^^38^^,^^40^^,^^54^ and perhaps it is this that contributed to their being among the most complained about members of the NHS workforce.^60^^,^^85^^,^^86^ In performing this role evidence suggests they receive only informal support from colleagues, and no training in coping with conflict.^87^^,^^88^ Since the pandemic, patient antipathy has become more sinister with increasing incidents of physical abuse,^18^^,^^89^ with some mainstream media outlets fuelling distrust between patient and primary care provider.^15^^,^^90^ There was evidence that the experience of receptionists can be improved by reducing cognitive load, improving training and feedback, and ensuring that IT systems harmonise with personnel and work practices.^29^ In other high-income countries including Australia there have been recent moves to improve satisfaction and workforce retention in part by ‘professionalising’ receptionists by increasing training and qualifications.^65^^,^^91^

### Implications for practice

As general practice adapts to the introduction of integrated care systems and associated collaborative working with secondary, community, and social care settings, the pressure on receptionists is set to continue.^57^ This review confirms their part as key non-clinical members of the practice team performing a series of clinically related tasks in a highly pressured environment. However, the additional complexity presented by broader system-wide integration offers the opportunity for policymakers and senior staff to reappraise their role for what it consists of: a multiplicity of administrative, organisational, patient-centred, and clinically responsible tasks. At a time when the ARRS is funding additional non-clinical roles in primary care to improve patient experience, developing a structured and holistic training programme for GP receptionists appears an obvious step.^92^ Competencies might include safe prescribing practices, literacy in medical terminology and result communication, or the ability to sensitively manage interactions with concerned or anxious patients. It is also worth acknowledging the overlap of the traditional signposting responsibility of receptionists with the new NHSE categorisation of that aspect of the work as ‘care navigator’, trained to direct patients to various sources of help, advocacy, and support.^31^^,^^32^ However, the recognition of the clinical influence of receptionists requires careful political and social consensus-building and brave commissioner-and policy-level decision making; it cannot be the responsibility of practice organisations with already stretched resources.^93^

Despite the evidence demonstrating receptionists’ impact on safe, effective care and patient experience, they continue to perform the role without mandatory or structured support from senior clinical colleagues or commissioners. With the growing, if controversial, integration of ARRS-funded non-clinical staff into primary care teams the time is right for policymakers, commissioners, and senior practice staff to reconsider the parameters of the role to include clinically relevant tasks, and explicitly recognise its value and the support and training needed to sustain it.

## Data

All studies included or referenced in this study are publicly available.
